# Transplacental transfer of RSV antibody in Australian First Nations infants

**DOI:** 10.1002/jmv.27383

**Published:** 2021-10-16

**Authors:** Nusrat Homaira, Michael Binks, Gregory Walker, Natasha Larter, Katrina Clark, Megan Campbell, Lisa McHugh, Nancy Briggs, Joyce Nyiro, Sacha Stelzer-Braid, Nan Hu, Kristine Macartney, Tom Snelling, Saad B. Omer, William Rawlinson, Ross Andrews, Adam Jaffe

**Affiliations:** 1 Discipline of Paediatrics, School of Women’s and Children’s Health, Faculty of Medicine and Health, UNSW, Sydney, New South Wales, Australia; 2 Department of Respiratory, Sydney Children’s Hospital, Randwick, UNSW, Sydney, New South Wales, Australia; 3 Menzies School of Health Research, Darwin, Northern Territory, Australia; 4 Prince of Wales Hospital, Sydney, New South Wales, Australia; 5 Sydney Children’s Hospitals Network, Sydney, New South Wales, Australia; 6 National Centre for Immunisation Research and Surveillance (NCIRS), Sydney, New South Wales, Australia; 7 Centre for Aboriginal Health, New South Wales Health, Sydney, New South Wales, Australia; 8 School of Public Health, University of Queensland, Brisbane, Queensland, Australia; 9 Stats Central, Mark Wainwright Analytical Centre, UNSW, Sydney, New South Wales, Australia; 10 KEMRI-Wellcome Trust Research Programme, Kilifi, Kenya; 11 University of Sydney, Sydney, New South Wales, Australia; 12 Yale Institute for Global Health, New Haven, Connecticut, USA; 13 Australian National University, Canberra, Australia

**Keywords:** indigenous infants, RSV antibody, transplacental transfer

## Abstract

Respiratory syncytial virus (RSV) is the leading cause of acute lower respiratory infection hospitalisations in Aboriginal infants specifically those aged <6 months. Maternally derived RSV antibody (Ab) can protect against severe RSV disease in infancy. However, the efficiency of transplacental transfer of maternal anti-RSV Ab remains unknown in Aboriginal infants. We characterised RSV Ab in Australian First Nations mother-infant pairs (*n* = 78). We investigated impact of covariates including low birthweight, gestational age (GA), sex of the baby, maternal age and multiparity of the mother on cord to maternal anti-RSV Ab titre ratio (CMTR) using multivariable logistic regression model. All (*n* = 78) but one infant was born full term (median GA: 39 weeks, interquartile range: 38–40 weeks) and 56% were males. The mean log_2_ RSV Ab titre was 10.7 (*SD* ± 1.3) in maternal serum and 11.0 (*SD* ± 1.3) in cord serum at birth; a ratio of 1.02 (*SD* ±0.06). One-third of the pairs had a CMTR of <1 indicating impaired transfer. Almost 9% (7/78) of the term infants had cord RSV Ab levels below <log_2_ 9. Covariates showed no effect on CMTR. Further mechanistic research is needed to determine the significance of these findings on RSV disease in First Nations children.

## 1 Introduction

Globally, acute lower respiratory infections (ALRIs) including bronchiolitis and pneumonia, are the leading causes of childhood morbidity and hospitalisation. Respiratory syncytial virus (RSV) alone is associated with 28% of all ALRI episodes in children aged <5 years old, making it the leading cause of paediatric respiratory hospitalisation.^1^ Most of the severe RSV disease leading to hospitalisation occurs in children aged <1 year old, particularly in the first 6 months of life.^1^

Protection of infants against severe RSV disease from birth to first 6 months of life, when they are most vulnerable, is generally thought to be augmented by high levels of maternally derived RSV-specific antibody (Ab). Higher levels of maternally derived RSV Ab in cord blood can extend the duration of protection against RSV disease in infancy and decrease disease severity.^2^ In American Indian infants, every unit increase in log_2_ RSV Ab titre in cord blood at birth was associated with a 30% reduced risk of RSV hospitalization in the first 180 days of life^3^

Cord blood to maternal blood RSV Ab titre ratio (CMTR) of ≥1 indicates efficient transfer.^4^ It has been documented that the extent of transfer of pathogen specific maternal Ab is influenced by total maternal immunoglobulin (immunoglobulin G [IgG]) levels, IgG sub-class, gestational age (GA) and certain underlying pathological factors including impaired placental integrity.^5^ Also transfer of maternal Ab occurs differently for different pathogens and disease specific transfer of Ab occurs predominantly during the third trimester of pregnancy and is therefore impacted by preterm birth.^6^

First Nations’ children worldwide are at 2–4 times increased risk of severe RSV disease and are likely to be younger when they develop their first episode of severe RSV disease compared to other children.^7^ Once hospitalised with severe RSV disease, First Nations children are more likely to require mechanical ventilation and remain hospitalized for longer periods (3–5 days) compared to non-indigenous children.^7^ Approximately 3% of the Australian population are Aboriginal and/or Torres Strait Islander (henceforth, respectfully referred to as Australian First Nations). Like First Nations’ children in other parts of the world, Australian First Nations children also suffer a significantly higher (two times) burden of hospitalisation associated with severe RSV disease compared to nonindigenous children, specifically in the first 6 months of life.^8^

Despite RSV being the main viral aetiology associated with highest burden of severe disease in infancy, there are no vaccine available against severe RSV disease. Palivizumab, a humanized monoclonal antibody (Ab), is the only currently available prophylactic therapy to protect against severe RSV disease in infancy.^9^ However, palivizumab is only recommended for high-risk children, including children born preterm or children born with chronic lung and heart disease but not specifically for Indigenous children.^10^

Fortunately, there are several vaccines and long-acting monoclonal antibodies that are in advanced stages of clinical trials. While a recent phase three clinical trial of a third-trimester dose of a maternal RSV vaccine to boost RSV Ab transfer to infants did not meet pre-specified primary efficacy criterion, RSV associated hospitalisation was reduced by 44%.^11^ These findings indicate that the vaccine might be beneficial in Indigenous infants and other high-risk populations where rates of RSV hospitalisations are exceptionally high. However, the efficacy of maternal vaccine is dependent on efficiency of transplacental transfer of maternal Ab from mother to infants. As such, an understanding of the immunological protection dynamics in different target populations is crucial. Further, pregnant Australian First Nations women have a higher risk of infection, preterm birth, gestational diabetes, anaemia, preeclampsia and other morbidities compared to non-Aboriginal mothers.^12^ In this complex context data on the prevalence and magnitude of RSV-specific Ab in Australian First nations’ mother-infant pairs are unknown.

The aim of this study was to measure RSV Ab levels in Australian First Nations mothers and their neonates at birth and determine the efficiency of transplacental transfer of RSV Ab.

## 2 Materials and Methods

During March-June 2020, we conducted a post hoc analysis of samples and clinical data collected during a randomised controlled trial of effectiveness of maternal immunization with 23-valent pneumococcal polysaccharide vaccine against middle ear disease in Australian First Nation infants in Northern Territory (NT) Australia.^13^ Australian First Nations women aged 17–39 years living in Darwin, Alice Springs and remote NT communities, between 2006 and 2011 and scheduled to have a singleton uncomplicated pregnancy at either Royal Darwin or Alice Springs Hospital were eligible to participate in the trial. Demographic and participant characteristic data were collected by parent interview at enrolment, birth, and infant study visits. Paired maternal (by study staff) and cord blood samples (by delivery suite staff) were collected from all participants at delivery. After centrifugation, serum samples were stored at -80°C in the Research Laboratory of Menzies School of Health Research, Darwin, NT. Each newborn was followed up prospectively until 7 months after birth and episodes of RSV-coded respiratory hospitalisations were identified from hospital medical records. There were 80 paired mother-infant serum samples available for further testing and were transported on dry ice to the Serology and Virology Division at Prince of Wales Hospital (POWH), Sydney. At POWH, neutralisation titres were measured by plaque reduction neutralisation test. Serial twofold dilutions of heat-inactivated serum (30 min, at 56°C, no complement added) were prepared in MEM (Cat #11090081; Gibco™, Life Technologies) supplemented with 1x PSG (Cat #10378016; Gibco™, Life Technologies), from 1:80 to 1:10240. The serial dilutions were incubated for 60 min at 37°C with an equal volume of an RSV-A isolate, for approximately 75 PFU/50 μl. A total of 50 μl of the serum-virus mixture was then added in triplicate to confluent Vero monolayers in 96-well flat-bottom tissue culture plates. The RSV-A isolate used were isolated and cultured from clinical specimen collected in 2019. The dilutions were incubated for 2 h at 37°C. Inoculum was replaced with 100 μl of infection overlay (MEM+ 2% FBS + 1x PSG+ 0.8% CMC), and plates were incubated for 48 h at 37°C (5% CO_2_). The infection overlay was removed, and Vero cells were fixed with 4% PFA. Fixed cells were immunostained by a 60 min incubation with mouse anti-F monoclonal primary antibody (Cat: MAB8599; Millipore) diluted to 1:2000 in phosphate-buffered saline (PBS), followed by 30 min with a goat anti-mouse IgG horseradish peroxidase-conjugated secondary antibody (Cat: ab205719; Abcam) diluted 1:500 in PBS. Plaques were developed with Dako AEC Substrate Chromogen (Cat: K3461; Agilent). After each step, wells were washed three times with PBS. Reading and counting of plaques was performed using an EliSpot Reader (Autoimmun Diagnostika GmbH).^14,15^ Paired maternal and cord sera were tested on the same microtiter plate alongside virus-only (no serum) and cell-only (no-virus) controls. The mean anti-RSV Ab titres were expressed logarithmically (log_2_). CMTR was calculated for each mother-infant pair. A CMTR of ≥1 was considered normal and CMTR<1 as impaired transplacental transfer.^16^ We also investigated the association of covariates including low birthweight (<2500 g), infants’ weight at birth, GA, sex of the baby, maternal age and multiparity of the mother on CMTR using multi-variable logistic regression model. RSV-coded hospitalisation data were analysed descriptively.

This study was approved by The Human Research Ethics Committee (HREC) of the NT Department of Health and Menzies School of Health Research (HREC 2019-3581).

## 3 Results

Eighty paired mother-infant samples were studied; in two of 80 samples (2.5%) the quantity of sera was insufficient for testing and were not included in the analysis. The median maternal age at birth was 24.7 years (interquartile range [IQR]: 21.4–28.7 years) and almost half (37/78) of the women reported of smoking during pregnancy. All but one of the 78 infants were born full term (median GA: 39 weeks, IQR: 38–40 weeks), 91% (71/78) had birthweight of ≥2.5 kg, 56% were males and 83% (65/78) were breast fed for at least 6 months of age.

**Figure 1:**
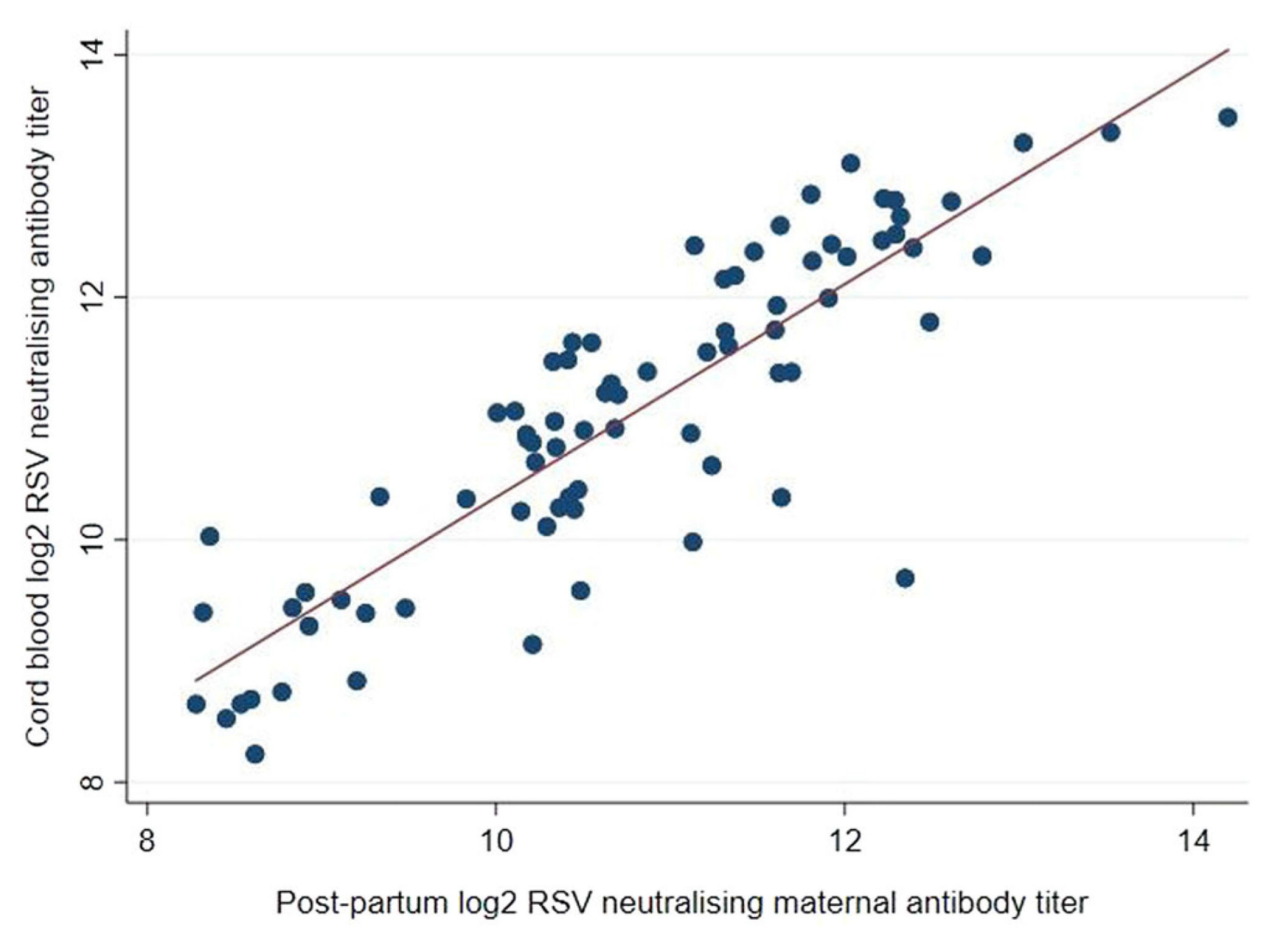
Respiratory syncytial virus neutralising antibody titres from 78 Australian First Nations mother-infant pairs

The mean log_2_ RSV Ab level in maternal blood at delivery was 10.7 (*SD* ±1.3) and in infant cord blood was 11.0 (*SD* ±1.3). Approximately 9% (7/78) of the infants had cord blood log2 RSV Ab level of <9. The mean CMTR was 1.02 (*SD* ± 0.06). Almost one-third (22/78) of the mother-infant pairs had CMTR < 1. Maternal and infant cord blood Ab titres at birth were correlated (*R* =0.87) (Figure 1). None of the covariates had an effect on impaired CMTR.

Eight infants (10%) developed nine episodes of RSV-coded hospitalisation in the first 6 months of life (median age at RSV hospitalisation was 3.9 months, IQR: 1.9–5.3 months) with one infant developing two episodes. The mean cord serum RSV Ab log_2_ titre was 10.7 (*SD* ± 0.3) in infants who had an episode of RSV-coded hospitalisation and 11.0 (SD ± 0.2) in those who did not. The CMTR was <1 in 37.5% (3/8) of the infants who had a RSV-coded hospitalisation vs 27% (19/70) in those who did not.

## 4 Discussion

To our knowledge this is the first study describing the efficiency of transplacental transfer of maternal RSV Ab level in Australian First Nations mother-infant pairs. The mean log RSV Ab titre and the CMTR in Australian First Nations infants were equivalent to levels in Alaska Native infants.^17^ Although we did not compare our results to non-Aboriginal mother-infant pairs, the study done in Alaska Native infants showed that levels of maternal RSV Ab level in Alaska Native infants was significantly lower than their comparator group who were predominantly white infants in United States (75 Alaska Native mother-infant pairs enrolled from Bethel, Alaska vs. 57 mother-infant pairs from Seattle). A similar trend could be observed in Australia and warrants exploration, based on the data from this study.

The CMTR observed in our study was comparable to other high-burden populations with similar socio-demographic risk factors ^18^ but there are many unique factors that are specifically linked to Fist Nations population which were not reflected in our study cohort. For example, as all the mother-infant pairs in this study were part of a vaccine trial whereby only pregnant women with uncomplicated pregnancies were recruited, all our infants were born at term and findings from our study do not represent the transfer efficiency of maternal RSV Ab in 14% of First Nations children who are born preterm (GA ≤36 weeks)^8^ and are more likely to have CMTR of <1. Nevertheless, in this cohort of term infants one-third of the mother-infant pairs had suboptimal CMTR indicating that there could be factors unique to Australian First Nations infants. Additionally, 9% of the infants had cord blood log_2_ RSV Ab level of <9. Although the exact level of RSV Ab that renders clinical protection is not known, it has been shown that log_2_ RSV Ab titres of 9 in infant cord blood or higher correlate with protection against severe RSV disease and every unit increase in log_2_ RSV Ab can reduce risk of RSV hospitalisation by 30%.^3^ Australian Aboriginal mothers have a higher prevalence of preterm births and maternal smoking during pregnancy compared to non-Aboriginal mothers, both of which are significant risk factors for severe RSV disease in infancy.^19^ Maternal smoking also effects placental vascularisation and is a significant risk factor for preterm births which in turn negatively impacts transplacental transfer of maternal Ab. However, how these factors interplay to impact the efficiency of transplacental transfer of maternal RSV Ab and RSV disease severity in Aboriginal infants remain uncharacterised.

This study had some limitations, we did not have serums samples from non-Aboriginal mother-infant pairs which limited our ability to compare levels of RSV antibody between Fist Nations and non-Aboriginal mother-infant pairs. We did not test maternal serum samples for total IgG levels and for specific pathogen specific antibody levels which limited our ability to infer the impact of maternal total IgG on transfer of RSV specific maternal Ab.^5^ We also had too few mother-infant pairs (n = 78) to have adequate power to investigate the role of maternally derived RSV Ab levels on the incidence of severe RSV disease in early infancy.

Further mechanistic studies are needed to elucidate the role of maternal RSV Ab in preventing severe RSV disease in Australian First Nation infants, and thereby inform future strategies, like the use of maternal vaccines and monoclonal antibodies, in this high disease burden population.

## Data Availability

The data are not publicly available due to privacy and ethical restrictions.

## References

[1] Shi T, McAllister DA, O’Brien KL (2017). Global, regional, and national disease burden estimates of acute lower respiratory infections due to respiratory syncytial virus in young children in 2015: a systematic review and modelling study. The Lancet.

[2] Chu HY, Steinhoff MC, Magaret A (2014). Respiratory syncytial virus transplacental antibody transfer and kinetics in mother-infant pairs in Bangladesh. J Infect Dis.

[3] Eick A, Karron R, Shaw J (2008). The role of neutralizing antibodies in protection of American Indian infants against respiratory syncytial virus disease. Pediatr Infect Dis J.

[4] Suara RO, Piedra PA, Glezen WP (1996). Prevalence of neutralizing antibody to respiratory syncytial virus in sera from mothers and newborns residing in the Gambia and inThe United States. Clin Diagn Lab Immunol.

[5] Chu HY, Englund JA (2014). Maternal immunization. Clin Infect Dis.

[6] Englund JA, Glezen WP, Thompson C, Anwaruddin R, Turner CS, Siber GR (1997). Haemophilus influenzae type b-specific antibody in infants after maternal immunization. Pediatr Infect Dis J.

[7] Singleton RJ, Petersen KM, Berner JE (1995). Hospitalizations for respiratory syncytial virus infection in Alaska Native children. Pediatr Infect Dis J.

[8] Homaira N, Oei J-L, Mallitt K-A (2016). High burden of RSV hospitalization in very young children: a data linkage study. Epidemiology & Infection.

[9] Group* I-RS (1998). Palivizumab, a humanized respiratory syncytial virus monoclonal antibody, reduces hospitalization from respiratory syncytial virus infection in high-risk infants. Pediatrics.

[10] Leclair JM, Freeman J, Sullivan BF, Crowley CM, Goldmann DA (1987). Prevention of nosocomial respiratory syncytial virus infections through compliance with glove and gown isolation precautions. N Engl J Med.

[11] Madhi SA, Polack FP, Piedra PA (2020). Respiratory syncytial virus vaccination during pregnancy and effects in infants. N Engl J Med.

[12] Comino E, Knight J, Webster V (2012). Risk and protective factors for pregnancy outcomes for urban Aboriginal and non-Aboriginal mothers and infants: the Gudaga cohort. J Maternal child health Journa.

[13] Binks MJ, Moberley SA, Balloch A (2015). PneuMum: impact from a randomised controlled trial of maternal 23-valent pneumococcal polysaccharide vaccination on middle ear disease amongst Indigenous infants, Northern Territory, Australia. Vaccine.

[14] Walker GJ, Naing Z, Ospina Stella A (2021). SARS coronavirus-2 microneutralisation and commercial serological assays correlated closely for some but not all enzyme immunoassays. Viruses.

[15] Sande CJ, Mutunga MN, Medley GF, Cane PA, Nokes DJ (2013). Group- and genotype-specific neutralizing antibody responses against respiratory syncytial virus in infants and young children with severe pneumonia. J Infect Dis.

[16] Atwell JE, Thumar B, Robinson LJ (2016). Impact of placental malaria and hypergammaglobulinemia on transplacental transfer of respiratory syncytial virus antibody in Papua New Guinea. J Infect Dis.

[17] Chu HY, Newman KL, Englund JA (2021). Transplacental respiratory syncytial virus and influenza virus antibody transfer in alaska native and seattle mother-infant pairs. J Pediatric Infect Dis Soc.

[18] Chu HY, Tielsch J, Katz J (2017). Transplacental transfer of maternal respiratory syncytial virus (RSV) antibody and protection against RSV disease in infants in rural Nepal. J Clin Virol.

[19] Homaira N, Mallitt KA, Oei JL (2016). Risk factors associated with RSV hospitalisation in the first 2 years of life, among different subgroups of children in NSW: a whole-of-population-based cohort study. BMJ Open.

